# 

**Published:** 1872-08

**Authors:** 


### INDEX.

### A

Abbott, F. W., M. D., Translation of Helmholtz on Recent Advances in Theory of Vision ...... 18, 57, 94
Albany County Medical Society. Proceedings of. Reported by F. C. Curtis, M. D., Secretary ...... 127, 208, 250, 331, 361, 413, 459
Albany County Medical Society. Thanks for Reports of ...... 1[illegible]5
American Medical Association—
Twenty-third Volume of Transactions, 198
Notice of Twenty-fourth Annual Meeting ...... 317
American Medical Association—
Report of Twenty-fourth Annual Session, 393
Anaplasia, Human, by mucous grafts from rabbits and oxen ...... 431
Anchylosis of Lower Jaw, with operations for its relief ...... 192
Appleton, D. & Co.'s, Catalogue ...... 154
Artificial Dilatation of the Anus. By. G. Simon, M. D ...... 427
Association of American Medical Editors, 347
Asthma, Dr. Williams' Treatment of ...... 148

### B

Bailey, James S., M. D., Stricture of Esophagus ...... 49
Bigelow, J. M., M. D., Rheumatic Peritonitis ...... 414
Boldo. A New Remedy ...... 193
Books and Pamphlets received—40, 79, 120, 159, 200, 240, 280, 320, 359, 400, 440, 472
Books Reviewed—
Anatomy, Hand-book of Morbid. By Francis Delafield, M. D. ...... 397
Angular Curvature of the Spine. By Benj. Lee, M. D ...... 76
Apoplexy, Cerebral Hemorrhage, etc. By John A, Liddell, M. D ...... 356
Autumnal Catarrh. By Morrill Wyman, M. D ...... 36
Blandford on Insanity ...... 35
Catalogue of Library of Surgeon-General's Office ...... 37
Cataract, New Method of Extraction of. By R. Liebreich, M. D ...... 199
Civil Malpractice. By M. A. McClelland, M. D ...... 438
Clinical Lectures on Diseases Peculiar to Women. By Lombe Atthill, M. D., 350
Connecticut School for Imbeciles, Annual Report of ...... 118
Contagious and Syphilitic Diseases. By John Morgan, M. D ...... 198
Doctor in Medicine. By Stephen Smith, M. D ...... 36
Diseases of the Throat. By J. Solis Cohen, M. D ...... 78
Diseases of Women, Diagnosis and Treatment of. By Grailly Hewitt, M. D ...... 239
Epidemic Cerebro Spinal Meningitis. By Meredith Clymer, M. D ...... 158
First Annual Report of the Supervising Surgeon of the U. S. Marine Hospital Service for 1872 ...... 358
Fistula, Hemorrhoids, Painful Ulcer, Stricture, Prolapsus, and other diseases of the Rectum. By Wm. Allingham, F. R. C. S ...... 351
Hallucinations of Childhood. By H. M. Knight, M. D ...... 117
Hand-book of Compound Medicines. By A. J. Cooley, M. D ...... 199
Hand-book for the Physiologial Labratory. By E. Klein and others ...... 471
Stricker ...... 319
Histology, A Manual of. By Prof. S.
Influence of the Mind upon the Body in Health and Disease. By D. Hack Tuke, M, D ...... 353
Lithotomy and Lithotrity. By Gurdon Buck, M. D ...... 77
Medical and Surgical History of the War of the Rebellion. Prepared under direction of the Surgeon-General ...... 357
Mental Pathology, contributions to By Isaac Ray, M. D ...... 355
Microscope and Microscopical Technology. By Heinrich Frey ...... 238
Nervous System, Diseases of. By Wm. A. Hammond, M. D ...... 197
Obstetric Aphorisms. By Joseph G. Swayne, M. D ...... 279
Obstetrics, theory and practice of. By Wm. H. Byford, M. D ...... 352
On Food. By H. Letheby, Ph. D ...... 115
Operative Surgery. By C. F. Maunder, 352
Oral Surgery. By James Garretson, M. D ...... 240
Ovarian Tumors, Diagnosis and Treatment of. By W. L. Atlee, M. D. 197
Ovarian Tumors, their Pathology, Diagnosis and Treatment. By E. Randolph Peaslee, M. D ...... 159
Ovaries, Diseases of, their Diagnosis and Treatment. By T. Spencer Wells ...... 349
Pharmacopœia of the United States ...... 351
Physical Diagnosis, Lessons in. By A. L. Loomis, M. D ...... 196
Physiology of Man. The Nervous System. By Austin Flint, Jr., M D., 115
Post Mortem Examinations and Morbid Anatomy. By Francis Delafield, M. D ...... 397
Proceedings of the Royal Society ...... 118
Qualitative Analysis, Manual of. By Robert Galloway, M. D ...... 78
Report on Practical Medicine. By T. D. Washburn, M. D ...... 118
Report to the Surgeon-General on the Anatomy of two cases of Cancer. By Asst. Surgeon J. J. Woodward ...... 37
Renal, Urinary and Reproductive Organs, Functional Diseases of. By D. C. Black, M. D ...... 158

Rodent Ulcer, anatomy and development of. By J. C. Warren, M. D.. 197
Science and Practice of Medicine. By Wm. Aitken, M. D ................ 155
Small-pox and Vaccination. By Carl Both ................................ 158
Surgery, the Science and Art of. By J. Eric Erichsen ................... 357
Surgery, Principles and Practice of. By F. H. Hamilton, M. D ........... 156
Surgery, the Practice of, By Thos. Bryant, F. R. C. S .................. 354
Surgery, System of. By S. D. Gross, M. D ............................... 75
Surgical Diseases of Infants and Children. By M. P. Guersant. Translated by R. J. Danglison, M D. 279
Syphilis, Treatment of, with Subcutaneous Sublimate Injections. By George Lewin, M. D ......... 157
Ten Laws of Health. By J. R. Black, M. D ............................... 117
Therapeutics, Handbook of. By Sidney Ringer, M. D ...................... 278
Thermic Fever or Sunstroke. By H. C. Wood, Jr., M. D ................... 116
Urethra, Stricture of. By F. N. Otis, M. D ............................. 199
Urinary and Renal Diseases. By Wm. Roberts, M. D ....................... 196
Urinary Organs, Diseases of. By John W. S. Gouley, M. D ................ 398
Uterus, Diseases and Displacements of. By E. N. Chapman, M. D .......... 114
Watson's Practice of Physic ............................................ 34
Wohler's Outlines of Organic Chemistry. By Rudolph Fettig and Ira Remsen ........ 278
Year-Book of Therapeutics By H. C. Wood, Jr., M. D ..................... 37
Bright's Disease, Etiology of. By F. C. Curtis, M. D ................... 401
Brush, E. N. Report of two cases of Ovariotomy by Enucleation by Dr. J. F. Miner ........ 164
Brush, E. N. Reports of Clinics of Prof. J. F. Miner, M. D., on Surgical Cases at the Sisters of Charity Hospital ..... 133
Items and Selections ................................................... 71, 107
Buffalo General Hospital, Surgical Cases at. By C. C. F. Gay, M. D ..... 88
Buffalo General Hospital, Examination for Appointment of Resident Physician ......... 154
Buffalo Hospital of the Sisters of Charity, Clinical Remarks of Prof. J. F. Miner, M. D., on surgical cases occurring in. Reported by E. N. Brush... 133
Buffalo State Asylum for Insane, Laying of Corner Stone ................ 74
Buffalo Medical Association, Abstract of Proceedings of, L. W. Harvey, M. D., Secretary ............ 4.0, 455

**C**

Carbolic Acid, Dry Gangrene following use of ........................... 142
Caldwell J. J., M. D., Essentials of Cooking ........................... 161
Cerebro-Spinal Meningitis .............................................. 15
Central New York Medical Association, Meeting of ....................... 195
Changing the Ethics of the Medical Profession .......................... 277
Children's Diseases and their Treatment, 139
Chloroform, Clinical Lecture on Death from. By J. Eric Erichsen, M. D ...... 101
Clinical Experience in Private Practice. By Z. C. McElroy, M. D ........ 321
Constitution, etc, of the Medical Association of Central New York ...... 38
Crumb, D. C., M. D., Tuberculous Discharge through a Sinus perforating the external walls of the thorax ......... 138
Curtis, F. C., M. D., Etiology of Bright's Disease ..................... 401
Curtis, F. C., M. D.. Reports of Proceedings of Albany County Medical Society, 127, 208, 250, 331, 361, 413, 459
Curtis, F. C., M. D., Report of Proceedings of Sixty-seventh Annual Meeting of New State Medical Society ......... 271
Chace, D. E., M. D., Report of Semi-Annual Meeting of Erie County Medical Society ......... 445

**D**

Death from Chloroform, Lecture on ...................................... 101
Death from Ether ....................................................... 110
Death from Stings of Bees .............................................. 109
Decision in a Suit of Malpractice. By H. F. Montgomery, M. D ........... 343
Definition, proper, of the word Cure as applied to Medicine ............ 66
De Zouche, J. E., M. D., Hospital Hygiene, 406
Diagnosis, Acquirement of Facility in .................................. 189
Digestion of Starch in Infancy ......................................... 346
Drugs. Inferior and Impure ............................................. 73
Dyspepsia, Functional, Anæmia and Chloeosis, Brown-Sequard on the new treatment for ........ 288

**E**

Eastman, H. N., M. D., Veratum Viride in Pulmonary Hemorrhage ........... 51
Eclecticism Defined .................................................... 141
Electric Cautery in Uterine Surgery, 172, 220, 262, 304
Electrical Instruments of Galvano-Faradaic Company ..................... 318
Electricity as a Therapeutical Agent. By G. W. Frion, M. D ............. 337
Erie County Medical Society, Semi-Annual Meeting. Reported by D. E. Chace, M. D., Secretary ......... 445
Embalming, the Process of .............................................. 468
Elegant Medicines ...................................................... 153
Enucleation of Uterine Fibroids ........................................ 430
Epidemic Cerebro-Spinal Meningitis. By Charles Forbes, M. D ............ 256
Epilepsy Cured by Bromide of Potassium and Sulphate of Atropia ......... 193
Erie County Medical Society ............................................ 236
Esophagus, Stricture of. By James S. Bailey, M. D ...................... 49
Ether, Case of Death from .............................................. 110
Excision of Joints, Ten cases of. By Alfred T. Livingston, A. B ........ 281
Eye, Foreign Body in Interior of ....................................... 191
Eye, Penetrating Gunshot Wound of. By J. W. Southworth, M, D ........... 9

**F**

Femur, Disease of, with recovery after amputation at the Hip Joint ..... 144
Forbes, Chas, M. D., Epidemic Cerebro-Spinal Meningitis ................ 256
Forensic Medicine. By Chasles B. Knowlton, M. D ........................ 81

G

Gastrotomy, Case of .......... 192
Gay, C. C. F., M. D., Surgical Cases at the Buffalo General Hospital .......... 88
Gay, C. C. F., M. D., Varicose Ulcers .... 375
Glycogen, Claude Bernard's discoveries of .......... 191
Grafting with Rabbit Skin .......... 106
Green Wall Paper, Beware of .......... 190

H

Hamlin, F. H., M. D., Thermometer in Disease .......... 41
Harcourt, L. A., M. D., Case of Instrumental Delivery. Case of Imperforate Anus .......... 124
Harcourt, L. A., M. D., Foreign Body Removed from Urethra .......... 170
Helmholtz, Recent Advances in Theory of Vision, Translated by F. W. Abbott, M, D ..........18, 57, 94
Hemorrhage, Pulmonary, Veratrum Viride in. By H. N. Eastman, M. D ...... 51
Hernia, Strangulated, Cure by Aspiration, 1C5
Homeopathic Dose of Chloral, Death from .......... 345
Horses, Epidemic Lexicography of ...... 143
Horses, The Epidemic Catarrh among .. 113
Hospital Hygiene. By J. De Zouche, M.D. 406
Hutchinson, W. F., M. D., Review of a Recent Suit of Malpractice .......... 290

I

Imperforate Anus, Case of, By L. A. Harcourt, M. D .......... 124
Insane, Buffalo State Asylum for. Laying of Corner Stone .......... 74
Instrumental Delivery, Case of. By L. A. Harcourt, M, D .......... 124
Intermittent Cerebro Spinal Meningitis. By George Niemeier, M.D .......... 6
Inversion of Uterus, Remarkable Case, with reduction after twenty-two years standing By Prof. Jas. P. White, M.D. Reported by A. T. Livingston .......... 1
Items and Selections. By E. N. Brush, 71, 107
Items in Brief .......... 436

K

Knowlton, Chas. B, M. D., Forensic Medicine .......... 81

L

Lea, H. C., Catalogue of Medical Books, 154
Lindsay & Blakiston's Catalogue ....154, 397
Livingston, Alfred T., A. B., Ten Cases of Excision of Joints .......... 281
Livingston, Alfred T., M. D., Case of Absence of Uterus and Consequent Amenorhœa .......... 441
Livingston, Alfred T., Report of Case of Reduction of Inverted Uterus. By Prof. J. P. White, M. D ..........
Lower Jaw. Anchylosis of with operation for its relief .......... 193
Lynde, U. C., M. D., Rubber Bandage ... 383

M

Male Fern, Fluid Extract of in Tape Worm .......... 428
Malpractice, A Recent Suit for. Reviewed by W. F. Hutchinson, M. D .......... 290
McElroy, Z. C. M D., Clinical experience in private practice .......... 321
Medical Law for the Masses .......... 152
Menstrual Disorders, their effect upon the Vascularity and Nutrition of the Intra-Ocular Structures .......... 28
Mowris, J. A., M. D., Enlarged Prostate, Vesical Catarrh and consequent Renal Disease .......... 121
Montgomery, H. F., M. D., Decision in a Suit of Malpractice .......... 343
Massachusetts Medical Society and the Homeopathists .......... 470

N

New York State Medical Society—
Notice of meeting of ..........195, 238
Proceedings at sixty-seventh annual meeting. Reported by F. C. Curtis, M. D .......... 271
Niemeier, George, M. D., Intermittent Cerebro-Spinal Meningitis .......... 6
Notices of Pamphlets and Periodicals—
Atlantic Monthly .......... 238
Archives of Ophthalmology and Otology .......... 79
Archives of Scientific and Practical Medicine. By C. E. Brown-Sequard, M. D .......... 154
Half Hour Recreations in Popular Science. By Dana Estes .......... 38
Locke's National Monthly .......... 155
New York Observer .......... 155
Notices of New Publications .......... 439
Physicians Visiting List for 1871. By Lindsay & Blakiston .......... 199
The Medical Record .......... 195

O

Obituary Notices—
Dr. E. P. Gray, of St. Joseph, Mo .... 39
Lieut. Reid T. Stewart, son of Dr J. L. Stewart, of Erie, Pa .......... 75
Prof James Hadley, of Yale College, 151
Dr. O. K. Parker, of Clarence, N Y., 151, 194
Dr. Uriah G. Bigelow, of Albany .... 318
Dr. Hiram Taber, of Marilla .......... 396
Dr. D. V. Stranahan, of Warren, Pa.. 435
Obstetrics in the Sandwich Islands. By Chas H Wetmore, M. D .......... 90
Oneida County Medical Society—
Correspondence concerning .......... 315
Explanation concerning resolutions presented to .......... 318
Opening of Annual Lecture Course of 1872-3, in Buffalo Medical College, Notice of .......... 75
Ovarian Cyst Multilocular, Ovariotomy and recovery .......... 31
Ovariotomy by Enucleation. Report of of two cases by Dr. J. F. Miner. By E. N. Brush .......... 1C4
Ovariotomy .......... 465

P

Pancreatic Emulsion in Wasting Disease of Children .......... 391
Pancreatic Juice as a Therapeutical Agent 425
Pennsylvania State Medical Society, Twenty-third Annual Session of .......... 119
Perspiration, Suppression of .......... 428
Poisoned Wound from Bite of Snake and Tarantula, and Sting of Scorpion and Centipede .......... 111
Popular Lectures on Medical Subjects ... 433
Prostate, Enlargement of, Vesical Catarrh and consequent Renal disease, By J. A. Mowris, M. D .......... 121
Public, competency of the. to judge respecting systems of medical practice By T. D. Washburne, M. D .......... 241
Puerperal Septicæmia .......... 4[illegible]9
Pyæmia, Synovial Membranes in .......... 69

**Q**

Quackery and Superstition ............ 347

**R**

Replantation of Teeth .................... 393
Retention of Urine depending upon Stricture. Gouley's management of ..... 145
Rheumatic Peritonitis. J. M. Bigelow, M. D. ...................... 414
Rochester, Prof. T. F., Utrum Horum Mavis ........................ 218
Rubber Bandage. By U. C. Lynde, M. D. 383
Rubber Bands in the Treatment of Fractures. By J. W. Southworth M. D.... 380
Rumored Prevalence of Childbed Fever.. 348

**S**

Scarlet Fever, Prof. G. T. Elliott on Treatment of ......................... 151
Seaman, H., M. D., Use of Placental Forceps through the Speculum ............ 340
Sewer Gas, Effect of ..................... 143
Southworth, J. W., M. D., Penetrating Gunshot Wound of the Eye ............ 9
Southworth, J. W., M. D., Rubber Bands in the Treatment of Fractures .......... 380
Spectacles, Invention of .................. 143
Spinal Irritation, Case of. By S. Weed, M. D. ................................ 201
Spleen, Case of Protrusion of through Abdomen. By W. H. Williams, M. D. 86
Stevens' Triennial Prize ............... 434
Story of Eight Children at one Birth..... 143
Stricture of Esophagus. By James S. Bailey, M. D. .......................... 49
Syphilis, Clinical Remarks upon Treatment of. By Willard Parker, M. D.... 386
Syphilis, Ricord's Distinction between Hard and Soft Chancres ............... 187
Syphilis, Ricord's Treatment of .......... 149
Small Pox, The Treatment of ............ 466

**T**

Thermometer in Disease. By F. Hamlin, M. D. ................................ 41
Thoracentesis, Remarks of Austin Flint, M. D., from a Report of Twenty Cases, 384
Toner Lectures ..................... 348, 435
Transfusion, Immediate, successful case of ...................................... 103
Translation, Dr. Abbott's of Helmholtz's Recent Advances in Theory of Vision . 114
Tuberculosis with Discharge of Pus externally through a Sinus perforating the Walls of the Thorax. By D. C Crumb, 138
Typhoid Fever, Treatment of Hemorrhage of Bowels in. By S. Weed. M. D. 10
The Influence of Instruction on Public Health ................................ 468

**U**

University of Buffalo, Medical Department:
  Opening of Lecture Course of 1872 3.. 75
  Introductory Lecture ............... 119
  Twenty-seventh Annual Commencement Notice of ..................... 277
Urethra, Foreign Body Removed from. By L. A. Harcourt, M. D ............. 170
Uric Acid, Injection of .................. 426
Use of Placental Forceps through the Speculum. By H. Seaman, M. D ....... 340
Utrum Horum Mavis. By Prof. T. F. Rochester, M. D .................... 218
Uterus, Case of Absence of and Consequent Amenorrhœa. By A. T. Livingston, M. D ........................ 441
Ulcers, The Treatment of in Charity Hospital, N. Y ......................... 464

**V**

Vacine Virus, A Supply of ............... 238
Varicose Ulcers. By C. F. Gay, M. D.... 375
Veratrum Viride in Pulmonary Hemorrhage. By H. N. Eastman, M. D.... 51
Vision, Translation of Hemholtz on Recent Advances in Theory of. By F. W.. Abbott, M. D ...................18, 57, 94
Volume Twelve ........................ 34

**W**

Washburn, T. D., M. D., Competency of the public to judge respecting medical systems and practice ................. 241
Weed, S., M. D., Case of Spinal Irritation, 201
Weed, S., M. D., Treatment of Hemorrhage of Bowels in Typhoid Fever.. 10
Wetmore, Chas. H., M. D., Obstetrics in the Sandwich Islands ................ 90
White, Prof. James P., Remarkable Case of Inversion of the Uterus, with reduction after twenty-two years' duration .. 1
Williams, W. H., M. D., Case of Protrusion of Spleen through the Abdomen... 86
Wood, Wm. & Co.'s Catalogue ........... 154
Worms Beneath Integument of Face and Scalp, Removal of. By Eugene Smith, M. D ................................ 189

### Books and Pamphlets Received.

A System of Surgery; Pathological, Diagnostic, Therapeutic, and Operative. By Samuel D. Gross, M. D., L.L. D., D. C. L., Oxon., etc. Illustrated by upwards of fourteen hundred engravings. Fifth edition, greatly enlarged and thoroughly revised, in two volumes. Philadelphia: Henry C. Lea, 1872.

A Manual of Qualitative Analysis. By Robert Galloway, F. C. S., etc. From the fifth re-written and enlarged London Edition. Philadelphia: Henry C. Lea, 1872.

Diseases of the Throat. A guide to the Diagnosis and Treatment of Affections of the Pharynx, Œsophagus, Trachea, Larynx, and Nares. By J. Solis Cohen, M. D., etc. Illustrated by one hundred and thirty-three engravings on wood. New York: Wm. Wood & Co., 1872. Buffalo: T. Butler & Son.

The Physiology of Man. Designed to represent the existing state of Physiological Science as applied to the Functions of the Human Body. The Nervous System. By Austin Flint, Jr., M. D., etc. New York: D. Appleton & Co., 1872. Buffalo: Martin Taylor.

Proceedings of the Royal Society. Vol. XX, Nos. 130 to 134, inclusive. Received through the Smithsonian Institute, Agency of Wm. Wesley, 28 Essex St., Strand, London.

The use of Pepsine in the Diarrhœa of Infants. By James S. Hawley, M. D., Brooklyn, N. Y.

A Nomenclature of Diseases, with the reports of the Majority and of the Minority of the Committee thereon. Presented to the American Medical Association, at the meeting held in Philadelphia, May, 1872.

Bossanges' Catalogue of Anatomy. Paris: Gustave Bossange, 16 Rue du Dix Decembre.

Twenty-ninth Annual Report of the Managers of the New York State Lunatic Asylum, for the year 1871.

Report on Practical Medicine, made to the Illinois State Medical Society, at the Annual Meeting, May, 1872. By T. D. Washburn, M. D., Hillsboro, Ill.

Case of Excessive Hypodermic use of Morphia. Three hundred needles removed from the body of an insane woman. Reported by Judson B. Andrews, M. D. (From American Journal of Insanity.)

Constitution and Standing Rules of the Philosophical Society of Washington.

Catalogue and officers of the University of Wooster, for the collegiate year, 1871-72.

Tenth Annual Announcement of the New York Medical College for Women, session of 1872-73.

